# Measuring service quality at an online university: using PLS-SEM with archival data

**DOI:** 10.1007/s11233-021-09071-7

**Published:** 2021-06-16

**Authors:** Jos M. C. Schijns

**Affiliations:** Faculty of Management, Department of Marketing & Supply Chain Management, Open Universiteit (OUNL), Heerlen, the Netherlands

**Keywords:** Student satisfaction, Service quality in higher education, Structural equation modeling, PLS-SEM, Archival data, E-learning

## Abstract

The aim of this study is to analyze, evaluate and validate the NSE (National Student Enquiry) as a service quality measure helping both higher education institutions (HEIs) and students in their decision making. Every year the Dutch foundation ‘Studiekeuze123’ sends out a survey (the NSE) to collect data on service quality regarding education at HEIs in the Netherlands. We used the 2019 NSE-data from the only e-learning university in the Netherlands, the Open Universiteit (OUNL), containing a sample of 1287 students. PLS-SEM was used to analyze a conceptual model in order to understand the service quality factors that promote students’ level of satisfaction and willingness to recommend the HEI. Overall, the findings reveal that the quality of the NSE is sufficient to be used for performance analysis. Nine out of twelve service components taken into account for the OUNL are found statistically significant affecting students’ satisfaction and willingness to recommend. The results help HEIs promoting and managing students’ perceptions of the quality of education and support students in their decision making process. Since many HEIs had to make a transition from onsite to online education within a short period of time, due to the Covid-19 pandemic, service quality became a major concern for HEIs. As online learning systems are expected to stay, analyzing the service quality of the OUNL as a reputed online HEI can help other HEIs getting their online learning systems on track.

## Introduction

Higher education (HE) acts as a major driver in economic competitiveness (Singh & Prasad, 2016) and is vital for the development of a country’s human capital (Annamdevula & Bellamkonda, 2016b; Gupta & Kaushik, 2018). The more and the better education, the more resistant and resilient a person, a nation or a civilization becomes. Quality of education is influenced by, and has an effect on, a number of stakeholders (Mahapatra & Khan, 2007; Srikanthan & Dalrymple, 2007) such as: providers of resources (e.g. public and private funding bodies), users of outputs, i.e. graduates (e.g. employers), and employees of the higher educational institution (HEI), e.g. academics and administrators. Students, however, are suggested to be the primary recipients of the service provided and are considered to be the most relevant stakeholder of HEIs (Abdullah, 2006; Annamdevula & Bellamkonda, 2016b; Bowden, 2011; Gremler & McCollough, 2002; Hill, 1995; Marzo-Navarro et al., 2005; Sander et al., 2000; Sultan & Wong, 2014). The customer-centric (or student-centered) perspective on the quality of education (Sultan & Wong, 2012), therefore, will be the central focus of our study.

Service quality in higher education from a students’ perspective has been examined and empirically tested in a number of studies. Chitty and Soutar (2004), for example, empirically tested the European customer satisfaction index (ECSI) for its applicability in a HE setting. Abdullah (2006) developed the HEdPERF scale as a measuring instrument of service quality specifically in the higher education sector. The PHEd measure is presented by Sultan and Wong (2010b) as a comprehensive performance-based service quality model applicable to HEIs. In their studies Senthilkumar and Arulraj (2011) respectively Annamdevula and Bellamkonda (2016b) developed and validated a service quality instrument for its applicability in HEIs in specifically India, called SQM-HEI and HiEduQual respectively. The HEDQUAL scale, developed by Icli and Anil (2014), is a measurement scale of service quality in higher education, particularly for MBA programs. Teeroovengadum et al. (2016) introduced the higher education service quality scale HESQUAL as a second-order factor model integrating both the functional and the technical aspect of higher education quality.

The SERVQUAL scale (Parasuraman et al., 1988, 1991) and the SERVPERF scale (Cronin & Taylor, 1992), however, have received most attention in literature on educational service quality (Brochado, 2009; Sultan & Wong, 2012). Though there is no doubt about the importance of service quality in higher education, there is no common consensus on the type and number of service quality dimensions nor how to measure service quality in a HE context (Clewes, 2003; Annamdevula & Bellamkonda, 2016a, b). There is, however, substantial evidence that service quality in an educational context has to be regarded as a multidimensional construct (Teeroovengadum et al., 2019) containing multi-item dimensions (Gupta & Kaushik, 2018).

Concluding, a variety of instruments have been developed to assess the quality of higher education. The NSE (National Student Enquiry), issued by the Dutch foundation ‘Studiekeuze123’ for the first time in 2010, is one of these instruments. It is a survey employed on a regular basis. The NSE is used not only by HEIs to evaluate and rank their educational quality, but also by potential students to support their buying decision process since choosing a HEI is regarded as an uncertain and high-risk decision (Sultan & Wong, 2013). The NSE, therefore, is considered a prime information source helping students, both nationally and internationally, in selecting a particular HEI. The NSE primarily measuring service quality of HEIs, therefore, has become a means of differentiating one HEI from others and is a relevant tool in the Dutch HE arena.

Despite the NSE is a national student survey that exists for over ten years and in the Netherlands has become an important quality measure for HEIs as well as tool in students’ buying decision process, the NSE is neither presented nor discussed so far in international academic literature focusing on service quality in a HE context. Our aim, therefore, is not to develop a new or adapted measure for quality assessment in the context of HEIs, but to present, analyze, and evaluate the NSE as an existing tool to measure students’ perceived service quality and to assess its effects on students’ satisfaction and willingness to recommend the HEI. This will be done in the context of a public, online university (i.e., the Open Universiteit in the Netherlands: OUNL). The OUNL is one of fourteen universities in the Netherlands but is the only online university. As many HEIs had to make a transition from onsite to online education within a short period of time, due to the Covid-19 pandemic, service quality became a major concern for HEIs since the conditions and characteristics of online education differ from those of the traditional face-to-face approach (La Rotta et al., 2020). The OUNL provides online learning for over 35 years and reached top-three positions with respect to student satisfaction for more than fifteen consecutive years. As online learning systems are expected to stay also after the Covid-19 pandemic is on its return, analyzing the service quality of the OUNL as a reputed online HEI can help traditional class-based HEIs getting their online learning systems on track.

This paper is organized as follows. In the next section we first embed the NSE in theory, which is opposite to the regular approach to extract the tool from theory. The characteristics and components of the NSE are linked to literature. Then, the methodology applied for this study is presented. In the results section we focus on testing the reliability, validity and applicability of the NSE measurement using PLS-SEM with archival data collected by ‘Studiekeuze123’. Finally, research conclusions are presented, implications identified, and limitations and directions for future research are highlighted.

## Literature review

Since we use secondary data collected through the NSE survey tool initiated by the ‘Studiekeuze123’ foundation we have to theoretically frame the tool and the type of data gathered. As Studiekeuze123 measures service quality in HE, student satisfaction and students’ willingness to recommend, we theoretically elaborate on these three concepts.

### Service quality in HE

Both, defining service quality in HE and its measurement are debated extensively in literature (Brochado, 2009; O’Neill & Palmer, 2004). As a result, there is hardly consensus, neither about the definition of service quality in a HE context, nor about how to measure service quality (Annamdevula & Bellamkonda, 2016a; Clewes, 2003; Sultan & Wong, 2010a).

Definitions of service quality in HE for example differ depending on the perspective taken, being either service quality as a result of the difference between expectations and performance based on the gaps model as presented by Parasuraman et al. (1988, 1991), or service quality in terms of the perception component alone, that is without comparing to expectations (Brochado, 2009; Abdullah, 2006).

In measuring service quality in HE a number of alternative instruments have been implemented and evaluated (e.g. Brochado, 2009; Gupta & Kaushik, 2018). Brochado (2009), for example, compares five alternative measures of service quality in HE. Though SERVQUAL, developed by Parasuraman et al. (1988, 1991), is the most popular scale in the HE setting (Gupta & Kaushik, 2018), Brochado (2009) concludes that both the SERVPERF scale and the HEdPERF scale (Abdullah, 2006) are best capable of measuring service quality in HE. SERVQUAL measures service quality in terms of the difference between expectations and performance perceptions using the gaps model as presented by Parasuraman et al. (1985, 1988, 1991). SERVPERF, HEdPERF and the PHEd measure (Sultan & Wong, 2010b), however, measure service quality without comparing performance to expectations. Many researchers now believe that a performance-based measure is an improved means of measuring service quality in HE (Abdullah, 2006; Cronin & Taylor, 1992, 1994; O’Neill & Palmer, 2004; Sultan & Wong, 2010a).

Regarding measuring service quality in HE, the NSE does not include performance perceptions to be compared with expectations as in the confirmation-disconfirmation paradigm (Brochado, 2009). The NSE follows the perception paradigm since it only includes the students’ perceptions of performance as a determinant of service quality. Therefore, the approach by NSE fits with Cronin and Taylor (1992) who argue that service quality is derived from perceptions of performance alone, and that a performance-based measure explains more of the variance in measuring service quality than a perceptions-minus-expectations measure (Cronin & Taylor, 1994). The superiority of perception-only measures over perception-minus-expectation measures in an educational setting is supported further by Li and Kaye (1998) and Dabholkar et al. (2000). The perception-only paradigm as applied in the NSE, therefore, is suggested to be a valid approach.

### Dimensions of service quality in HE

Not only alternative instruments measuring service quality in HE have been studied empirically and conceptually, also the dimensions of service quality in HE have been studied (e.g. Gupta & Kaushik, 2018). Gupta and Kaushik (2018: 580) noticed “a huge variation in the items as well as constructs while exploring the dimensions”, “moving from a simple uni-dimensional construct to complex multidimensional constructs” (page 592). In their extensive literature review Gupta and Kaushik (2018) identified a range of three up to and including twenty-two dimensions of service quality in HE, the modus being five dimensions. Sultan and Wong (2012) identified a minimum of three, a maximum of eight and a modus of five service quality dimensions after investigating fifteen studies in HE.

The survey instrument called NSE includes a total of nineteen dimensions of the service quality concept. Twelve of them are applicable to the OUNL, the only university in the Netherlands offering distance learning as its core activity. Since the OUNL is (1) an academic university as opposite to a university for applied sciences, is (2) a university for distance learning only, and (3) the main language is Dutch, seven dimensions are not applicable. Table 1 shows all nineteen dimensions of the service quality concept included in the NSE (as of 2019) and those twelve applicable to the OUNL.

**Table 1:** Dimensions of service quality included in the NSE (2019) and those applicable to the OUNL (+)

	NSE: dimensions of service quality	#items NSE	OUNL	#items OUNL	Sample items “How satisfied are you with …”
1	Content and structure of study	10	+	10	The content quality of the study materials
2	Acquired general skills	6	+	6	Justifying your conclusions
3	Acquired scientific skills	5	+	5	Analytical thinking
4	Acquired skills for applied research	5	n.a.		
5	Preparation for professional career	3	n.a.		
6	Connection to professional field	3	+	3	The practical orientation of your training
7	Professors/Lecturers	9	+	7*	The didactic skills of teachers
8	Academic guidance/counselling	3	+	3	The quality of the guidance provided
9	Testing and assessment	5	+	5	The quality of practical examinations
10	Information provided	4	+	4	The information about your study progress
11	Program schedules	4	n.a.		
12	Study load	5	+	5	The possibility to combine working and learning
13	Group size	3	n.a.		
14	Internships	2	n.a.		
15	Internship experience	3	n.a.		
16	Study facilities	7	+	4**	The digital learning environment
17	Quality care	4	+	4	The way in which your study program uses the results of educational evaluations
18	Challenging character of study	4	+	4	The extent to which your study program challenges you to get the best out of yourself
19	Internationalization	4	n.a.		
	Total number of items	89		60	
*Note:* +: applicable; n.a.: not applicable; *: Two items excluded due to high proportion of missings; **: Three items excluded since they are not applicable for distance learning (e.g. the number of seats, availability of working stations, and physical library)					

Sultan and Wong (2010b, 2012, 2013, 2014) suggest that service quality models in HE should include “the three critical aspects of service quality, academic, administrative and facilities” (Sultan & Wong, 2013: 78). In Appendix 2 Table 7, we illustrate how these three critical aspects of service quality are captured by the performance-only service quality models developed for the measurement of perceived service quality specifically in the higher education sector, i.e.: HEdPERF, PHEd, HESQUAL, and NSE. Despite the variation in items and number of dimensions we conclude that the main service quality aspects academic, administrative and facilities are covered by the NSE.

### Student satisfaction

In line with the student perspective on service quality in education, satisfaction is also defined from the perspective of the student. Students’ perspective is central since students are the primary recipients of the service provided and, therefore, are considered to be the most relevant stakeholder of HEIs (Annamdevula & Bellamkonda, 2016b; Bowden, 2011; Gremler & McCollough, 2002; Hill, 1995; Marzo-Navarro et al., 2005; Sander et al., 2000; Sultan & Wong, 2014). Student satisfaction can be defined as “the favorability of a student’s subjective evaluation of the various outcomes and experiences associated with education” and “is being shaped continually by repeated experiences in campus life” (Elliott & Shin, 2002: 198).

In the NSE student’s satisfaction is driven by a student’s general assessment of “a web of interconnected experiences” associated with the HEI (Elliot and Shin 202: 198) and, therefore, is a cumulative concept (Teeroovengadum et al., 2019) measured through a multi-item scale and suggested to be a global or overall measure of satisfaction. Assessing overall student satisfaction using a composite satisfaction scale is suggested “to have more diagnostic value for strategic decision making” (Elliott & Shin, 2002: 207), which at the end is the main goal of Studiekeuze123 to distribute the NSE.

### Willingness to recommend

Customer loyalty is described as “a deeply held commitment to rebuy or repatronize a preferred product/service consistently in the future, thereby causing repetitive same-brand or same brand-set purchasing, *despite* situational influences and marketing efforts having the potential to cause switching behavior” (Oliver, 1999: 34). Based on this definition customer loyalty contains an attitudinal component and a behavioral component (Baldinger & Rubinson, 1996; Hennig-Thurau et al., 2001; Koslowsky, 2000; Marzo-Navarro et al., 2005).

Loyalty, however, “might not be an appropriate consequence in the context of higher education; instead, behavioural intention may play a vital role” (Sultan & Wong, 2013: 79; Sultan & Wong, 2014). As such, the NSE meets this observation since it measures students’ loyalty through a behavioral intention (i.e., the willingness to recommend the HEI) as a reflection of the attitudinal component of the loyalty concept. According to the theory of reasoned action, behavioral intentions are very accurate predictors of corresponding behavior (Ajzen & Fishbein, 1980; Fishbein & Manfredo, 1992).

### Integrated model including service quality, satisfaction and loyalty

The number of studies that examined integrated models of service quality in a HE context is limited. Sultan and Wong (2012) provide an overview of major studies and conclude that, overall, students’ satisfaction and loyalty are the main target variables. Perceived service quality has been found to be the critical determinant of satisfaction in different contexts (e.g., Carlson & O’Cass, 2010; Cronin & Taylor, 1992; Gounaris et al., 2010; Parasuraman et al., 1985, 1988; Schijns et al., 2016) including a HE context (e.g., Hasan et al., 2008; Alves & Raposo, 2007; Annamdevula & Bellamkonda, 2016a, b; Dehghan et al., 2014; Guolla, 1999; Ham & Hayduk, 2003; Sultan & Wong, 2012, 2013). Satisfaction is suggested to be an essential step in loyalty formation (Oliver, 1999). Student satisfaction has been found to be the crucial mediator in the effects of service quality on student loyalty in general (Annamdevula & Bellamkonda, 2016a, b; Dehghan et al., 2014) and on students’ willingness to recommend the institution in particular (Al-Alak, 2006; Athiyaman, 1997; Marzo-Navarro et al., 2005; Sultan & Wong, 2013, 2014).

Synthesizing the results of studies as exposed in the preceding discussion, the following hypotheses are put forward.

- H1.(1,n): (Each component of) The HEI’s service quality, as perceived by the students, has a significant positive effect on students’ overall satisfaction (n = the number of service quality components taken into account).
- H2: Students’ overall satisfaction has a significant positive effect on the willingness to recommend the HEI.

**Fig. 1:**
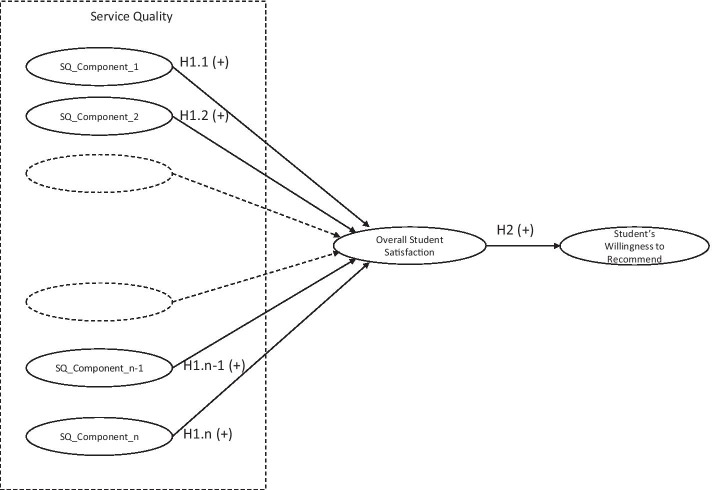
Conceptual model

In summary, the present study examines an integrated model where service quality is conceptualized on a perception-only measure (the NSE), containing multiple service quality dimensions, each having a positive effect on students’ overall satisfaction which subsequently results in positive word of mouth which has been modelled as the final consequence. Figure 1 shows our conceptual model.

## Methodology

### Sampling method

The online survey technique is the sampling method used by Studiekeuze123 to collect data from students. All Dutch HEIs are invited to participate in the NSE. The participating HEIs send in the e-mail addresses of their students. In January 2019 the students were invited to participate in the online NSE. It’s a user-friendly survey that can be completed through several devices (desktop, tablet, smartphone). After the initial invitation six reminders were sent, since response fell behind. The NSE ultimately was closed in April 2019. The overall response rate for 2019 is 30.2% (Studiekeuze123, 2019) which is consistent with similar studies testing and evaluating service quality models in HE (e.g., Sultan & Wong, 2010b).

### Sampling size

The NSE database captures data of 2,344,266 respondents, studying at a total of about ninety HEIs in the Netherlands, and collected from 2010 up to and including 2019. Due to the Covid-19 pandemic the survey for 2020 has been cancelled.

In the 2019 NSE survey thirty-two HEIs participated, generating data from 93,874 respondents (Studiekeuze123, 2019). The OUNL is one of the thirty-two HEIs that participated in het 2019 NSE. Studiekeuze123 invited 7655 OUNL-students to participate in the 2019 NSE survey, representing 53% of the OUNL student population (Open Universiteit, 2020). 1955 Students responded, a gross response rate of 25.5%. Ultimately, after profound data screening and cleaning 1287 usable records were included for further analyses in our study (a net response rate of 16.8%).

### Sampling profile

For privacy reasons the dataset as provided by Studiekeuze123 hardly includes background information about the respondents. Most background information refers to the HEI itself (code, name, location, etc.). Information about student’s educational program and stage (bachelor/master) is available. Student’s stage is used as a controlling variable in our analyses. 663 students (52%) are in the bachelor stage, 624 students (48%) are in the master stage. Students belong to various departments and schools across the HEI as shown by Table 2.

**Table 2:** Students divided per educational program and stage (bachelor/master)

Educational Program	Bachelor (52%)	Master (48%)
Humanities	114 (17%)	
Business Administration	46 (7%)	
Computer Science	51 (8%)	
Information Science	9 (1%)	
Environmental and Natural Science	33 (5%)	
Psychology	259 (39%)	
Law	151 (23%)	
Business Process Management and IT		131 (21%)
Computer Science		4 (1%)
Environmental Science		19 (3%)
Art & Humanities		27 (4%)
Management		215 (35%)
Educational Sciences		90 (14%)
Psychology		67 (11%)
Law		46 (7%)
Software Engineering		25 (4%)
	663 (100%)	624 (100%)

### Measures

#### Service quality

The NSE includes eighty-nine items measuring service quality in education. Not all these eighty-nine items are applicable to the OUNL (see Table 1) since the OUNL is a distance university located in the Netherlands having the largest number of off-campus students. Therefore, service aspects referring to, for example, the number of seats, availability of working stations, meeting rooms or physical library are not applicable. As a result, the perceived service quality construct in our study includes sixty items, covering twelve service quality dimensions, including academic, administrative and facility services (Sultan & Wong, 2013, 2014). All twelve service quality dimensions are conceptualized as formative measures. Table 1 includes a sample item for each of the service quality dimensions applicable to the OUNL.

#### Satisfaction

Twelve items were used to measure students’ overall satisfaction construct in our study. The twelve items reflect the twelve dimensions of service quality as applicable to the university at study. Satisfaction, therefore, is also conceptualized and operationalized as a formative measurement model. Table 3 provides some insight in the twelve items for measuring students’ overall satisfaction.

**Table 3:** Satisfaction items

How satisfied are you with … (1 = very dissatisfied; 5 = very satisfied)	
1. The content of the educational program	
2. Acquired general skills	
3. Acquired scientific skills	
4. Preparation for professional career	
5. Professors/Lecturers	
6. Information provided	
7. Study facilities	
8. Testing and assessment	
9. Study load	
10. Academic guidance/counselling	
11. Quality care	
12. Challenging character of study	

#### Willingness to recommend

To measure student loyalty, a single-item scale was used, capturing students’ willingness to recommend the HEI. Item wording: “Would you recommend your educational program to friends, family or colleagues?”

All the items for service quality and overall satisfaction were measured on a symmetric and equidistant five-point Likert scale ranging from 1 (very dissatisfied) to 5 (very satisfied). Willingness to recommend was also measured on a five-point Likert scale, but now anchored at (1) ‘No, absolutely not’ and (5) ‘Yes, absolutely’.

### Method of analysis

Since our main goal is to present, analyze, and evaluate the NSE in an attempt to identify key drivers for students’ satisfaction and willingness to recommend, a PLS-SEM approach is applied. Additional reasons to prefer PLS-SEM over CB-SEM (covariance-based SEM) are that our model includes many indicators (73 in total) as well as many (i.c. 13) formatively measured constructs. Also, a lack of normality as indicated by our data screening favors the use of PLS-SEM (Ghasemy et al., 2020).

## Results

For our empirical analyses we used a dataset containing 1287 qualified records extracted from the NSE database. The results are based on the final operationalization used in this study. We start with assessing the results of the measurement models followed by the analysis of the structural model as suggested by Hair et al. (2017).

### Results of the measurement model analyses

All measurement models, except the single-item scale for willingness to recommend, are formative in nature. We, therefore, follow the formative measurement models assessment procedure as described by Hair et al. (2017).

First we assess convergent validity doing a redundancy analyses for each formative construct. The NSE contains global, single-item measures of all twelve constructs that we used in the redundancy analyses. All path coefficients between the formative constructs and their global single-item equivalents are above the recommended threshold value of 0.70 (minimum of 0.802), suggesting that all formatively measured constructs show convergent validity.

Next, we assess our formative measurement models for collinearity issues by inspecting the outer VIF values (Variance Inflation Factors). VIF values range from a minimum of 1.029 (Testing_and_Assessment_06) to a maximum of 4.749 (Testing_and_Assessment_04). The outer VIF values of all items, therefore, are below the threshold value of 5 (Hair et al., 2017), suggesting that collinearity is not an issue with regard to our formative measurement models.

Third step is to assess the significance and relevance of the formative indicators. First, we test the significance of the outer weights. From Appendix 1 Table 6 we conclude that all formative indicators are significant at the 5% level, except the following six indicators: ‘General satisfaction_03’; ‘Professors/Lecturers_02’; ‘Content and structure of study_03’; ‘Content and structure of study_10’; ‘Quality care_02’; ‘Testing and assessment_06’. Of these six formative indicators, only ‘Testing and assessment_06’ has a loading less than 0.5 (i.e., 0.174). However, all loadings including the loading for ‘Testing and assessment_06’ (t-value = 2.970; *p* value = 0.003; 95% BCa [0.057–0.285]) turn out to be significant. We, therefore, retain all formative indicators.

Since our results indicate that our measurement models are sufficiently valid and reliable we proceed with analyzing our structural model.

### Results of the structural model analyses

First, we assess our structural model for collinearity issues. Table 4 shows the values for the inner VIFs. Since all inner VIF values are below the threshold value of 5, collinearity between the constructs is not a major issue (Hair et al., 2017).

**Table 4:** Inner VIF values, R^2^ values and f^2^ effect sizes

	Inner VIF values		R^2^-value (adjusted)	f^2^ effects for General satisfaction
	General satisfaction	Willingness to recommend		
Connection to professional field	2.160			0.013
General satisfaction		1.002	0.887 (0.886)	
Acquired general skills	3.929			0.000
Professors/Lecturers	3.658			0.128
Information provided	2.639			0.000
Content and structure of study	4.864			0.252
Quality care	2.959			0.006
Academic guidance/counselling	3.355			0.021
Study facilities	2.210			0.002
Study load	2.239			0.020
Testing and assessment	2.803			0.021
Willingness to recommend			0.506 (0.506)	
Challenging character of study	3.900			0.010
Acquired scientific skills	3.172			0.008

Table 4 also includes the R^2^ values for the endogenous latent constructs in our model: general satisfaction and willingness to recommend. The R^2^ value of willingness to recommend (0.51) can be considered moderate, whereas the R^2^ value of general satisfaction (0.89) can be described as substantial (Hair et al., 2017).

The f^2^ effect sizes are also included in Table 4. The f^2^ values suggest that content and structure of study has a large effect (0.252) on general satisfaction. Professors/Lecturers have a medium effect (0.128) on general satisfaction. Academic guidance/counselling (0.021), study load (0.020), and testing and assessment (0.021) all three have a small effect on general satisfaction. All other exogenous constructs are suggested to have no effect on general satisfaction since their f^2^ effect sizes are less than 0.02 (Hair et al., 2017).

### Hypothesis testing results

Table 5 shows the relevant path coefficients (hypotheses) and their significances. Hypotheses 1.1–1.12 refer to the effects of the twelve service quality components on students’ overall satisfaction. Hypothesis 2 refers to the effect of students’ overall satisfaction on the willingness to recommend the HEI.

**Table 5:** Hypothesis testing results

Hyp.		Path coefficient	T statistics	*P* values	95% BCa confidence interval		Hypothesis supported?
H1.1	Connection_to_professional_field - > General_satisfaction	0.056	3.574	0.000	0.027	0.088	Yes
H1.2	Acquired_general_skills - > General_satisfaction	−0.004	0.168	0.867	−0.051	0.037	No
H1.3	Professors/Lecturers - > General_satisfaction	0.230	7.896	0.000	0.173	0.286	Yes
H1.4	Information_provided - > General_satisfaction	0.011	0.591	0.554	−0.028	0.045	No
H1.5	Content_and_structure_of_study - > General_satisfaction	0.372	12.639	0.000	0.316	0.432	Yes
H1.6	Quality_care - > General_satisfaction	0.044	2.192	0.028	0.004	0.083	Yes
H1.7	Academic_guidance/counselling - > General_satisfaction	0.089	3.951	0.000	0.047	0.134	Yes
H1.8	Study_facilities - > General_satisfaction	0.022	1.209	0.227	−0.013	0.056	No
H1.9	Study_load - > General_satisfaction	0.071	3.340	0.001	0.031	0.113	Yes
H1.10	Testing_and_assessment - > General_satisfaction	0.082	3.967	0.000	0.043	0.124	Yes
H1.11	Challenging_study - > General_satisfaction	0.065	2.862	0.004	0.021	0.110	Yes
H1.12	Acquired_scientific_skills - > General_satisfaction	0.054	2.666	0.008	0.015	0.093	Yes
H2	General_satisfaction - > Willingness_to_recommend	0.712	38.001	0.000	0.669	0.744	Yes

Nine of the twelve service quality components significantly impact students’ overall satisfaction (Hypothesis 1). ‘Acquired general skills’, ‘Information provided’ and ‘Study facilities’ do not affect ‘General satisfaction’. In the specific context of the OUNL, ‘Content and structure of study’ (0.372), ‘Professors/Lecturers’ (0.230), and ‘Academic guidance/counselling’ (0.089) emerge as top three satisfaction drivers among OUNL students. ‘General satisfaction’ has a significant impact on ‘Willingness to recommend’ (Hypothesis 2).

### IPMA - results

In order to provide policy makers and university managers with actionable results, the data are also analyzed based on the importance-performance paradigm (Ghasemy et al., 2020; Martilla & James, 1977; Slack, 1994). The importance-performance map analysis (IPMA) assumes that evaluation criteria students use vary in importance. Importance ratings enable universities “to identify key drivers of student satisfaction and help them set the priorities for improvement efforts” (Elliott & Shin, 2002: 201). The IPMA, therefore, is of practical value for HEI management as it is “a means of both assessing and directing continuous quality improvement efforts within this sector” (O’Neill & Palmer, 2004: 49).

**Fig. 2:**
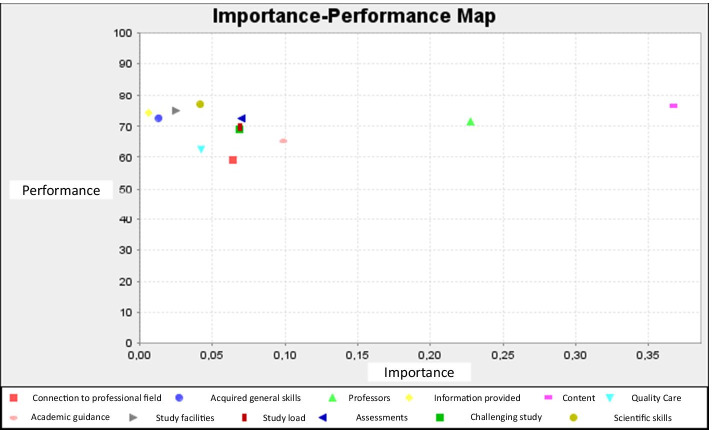
Importance Performance Map Analysis (IPMA)

In predicting overall student satisfaction the importance dimension of a predecessor service quality construct (e.g., content and structure of study; (the didactic skills of) Professors and lecturers; acquired scientific skills; study load) is represented by its total effect. The performance dimension of a predecessor service quality construct is represented by its average latent variable score. Both dimensions, importance (total effects) and performance (average latent variable scores), are provided by the SmartPLS 3 software (Hair et al., 2018; Ringle & Sarstedt, 2016) and, therefore, do not have to be asked by means of a questionnaire. From the IPMA results, shown in Fig. 2, we find that the service quality construct ‘content and structure of the study’ has a relatively high importance (strong total effect) for predicting overall student satisfaction. ‘Information provided’, as a predecessor, has a relatively low importance (weak total effect). The performance levels (the average latent variable scores, rescaled on a range from 0 to 100) range from 60 to 80.

The IPMA results as shown in Fig. 2 support earlier findings based on our f^2^ analyses and analysis of the path coefficients: for the OUNL, ‘Content and structure of study’, ‘Professors/Lecturers’, and ‘Academic guidance and counselling’, in that order, emerge as top three satisfaction drivers among OUNL students. The IPMA analysis also shows the relative high and comparable levels of performance on all service dimensions, regardless their level of importance. That is, the university performs well on each service dimension despite its importance.

## Conclusions, discussion and implications

### Conclusions and discussion

Testing and validating the service quality model for HEIs as used by the Dutch foundation Studiekeuze123 shows that the NSE instrument is robust and capable of measuring service quality in a HEI context from students’ perspective.

Our study reveals that the dimensions of service quality taken into account are nomological valid and show adequate reliability and validity supporting the findings of Brenders (2013). Nine out of twelve service quality components positively impact students’ satisfaction (Hypothesis 1). Our findings support previous research indicating perceived service quality is a critical determinant of satisfaction in a HE context (e.g., Hasan et al., 2008; Alves & Raposo, 2007; Annamdevula & Bellamkonda, 2016a, b; Dehghan et al., 2014; Guolla, 1999; Ham & Hayduk, 2003; Sultan & Wong, 2012, 2013).

In our study ‘Content and structure of study’ and ‘Professors/Lecturers’ by distance are the most important satisfaction drivers among OUNL students, followed by ‘Academic guidance and counselling’, ‘Testing and assessment’ and ‘Study load’. Content and structure as well as (interaction with) lecturers and faculty were also found by Ehlers (2004) and Peng and Samah (2006) as important service quality factors in the context of e-learning. Students as the primary recipients of the service provided want value for money and, therefore, expect high-quality content in exchange for the fees they pay. In their process of development, growth and becoming employable, students have to cooperate with professors and lecturers. Students and faculty are the co-producers of education and, therefore, students depend on faculty in successfully completing their studies.

The IPMA analysis also shows the relative high and comparable levels of performance on all service dimensions, regardless their level of importance. That is, the OUNL performs well on each service dimension independent of its importance. This observation might explain why this particular university has been positioned in the top-three rankings of Dutch universities for over fifteen years: the university scores consistently high on all service quality dimensions. The university cannot afford underperforming on any of the service quality dimensions, in order to hold its high quality standards and top-three ranking.

Based on the R^2^ values and f^2^ effects sizes the results also demonstrate a strong predictive ability of satisfaction and willingness to recommend. The R^2^ values of willingness to recommend (0.51) and general satisfaction (0.89) can be considered moderate respectively substantial.

The present study also shows that students’ overall satisfaction impacts the willingness to recommend the HEI (Hypothesis 2), supporting previous research that found student satisfaction to be a key predictor for student loyalty in general (e.g., Annamdevula & Bellamkonda, 2016a, b; Dehghan et al., 2014) and for the willingness to recommend the HEI in particular (e.g., Al-Alak, 2006; Athiyaman, 1997; Marzo-Navarro et al., 2005; Sultan & Wong, 2010a, b, 2013).

Overall, we conclude that the NSE is nomological sound and theoretically grounded as well as an empirically supported measure of service quality in HE. By applying our study in an online HE context we contribute to the limited research on identifying service quality factors in an e-learning setting (La Rotta et al., 2020; Uppal et al., 2018). Our model and results provide us with both theoretical and practical insights.

### Theoretical implications

Theoretical contributions are made in several ways. This study empirically examined the applicability of the National Student Enquiry (NSE) by validating constructs such as perceived service quality, student satisfaction and behavioural intention for higher education institutions. The NSE has been developed and improved during more than a decade and turns out to be “a reliable and valid instrument for measuring service quality of higher education from the students’ perspective”, a necessity when service quality is to be improved (Teeroovengadum et al., 2016: 245).

Service quality in HE is to be conceptualized as a multidimensional concept containing multi-item dimensions as suggested by a vast majority of literature (e.g., Gupta & Kaushik, 2018; Teeroovengadum et al., 2019) and supported empirically by our study. By capturing a total of 19 dimensions, including the three critical service quality aspects academic, administrative and facilities, the NSE measure reflects a holistic approach to service quality in HE (Teeroovengadum et al., 2016).

In the past the NSE has been tested and validated mainly in a face-to-face modality (Brenders, 2013). Uppal et al. (2018) and La Rotta et al. (2020), however, conclude that there is an increasing need to effectively assess service quality of HE in online settings, but that limited research was found in the literature. Since our study examined and validated the NSE particularly under an online modality we contribute to the scarce literature available addressing this issue.

Our study demonstrates and examines an integrated model including perceived service quality, satisfaction and loyalty in a HE setting. Our empirical findings support the positive relationships between perceived service quality and student satisfaction, and between student satisfaction and behavioural intentions (e.g., Al-Alak, 2006; Annamdevula & Bellamkonda, 2016a, b; Athiyaman, 1997; Dehghan et al., 2014; Marzo-Navarro et al., 2005; Sultan & Wong, 2010a, b, 2013).

Bearing on previous point, a service quality measure following the performance-perception paradigm is very appropriate when more comprehensive and integrated service quality models in HE need to be tested (e.g., Dabholkar et al., 2000; Li & Kaye, 1998).

The NSE, as a result, provides a useful tool for managers improving service quality in HEIs and for researchers building more comprehensive and robust models. We will elaborate more on both in following sections.

### Managerial implications

This study focused on presenting and evaluating the NSE measure and identifying key drivers for students’ satisfaction and willingness to recommend analyzing archival data from an online university in the Netherlands (the OUNL) using PLS-SEM. The current results show that the NSE is an appropriate and practical measurement instrument of service quality in a HE context supporting HEIs in promoting students’ satisfaction and willingness to recommend in an attempt to obtain advantage in a competitive environment.

Further, Studiekeuze123, provides data for a lot of HEIs in the Netherlands and from 2010 up to now. This offers the opportunity for not only benchmarking between HEIs, but also analyzing their development over time applying a longitudinal study. Since for many HEIs the number of respondents seem to be high enough the same approach and method can be used for internal benchmarking, e.g. between different educational programs at the same HEI. Hennig-Thurau et al. (2001), for example, found major differences between students from different educational programs in terms of the most relevant dimensions of service quality. In general, through the NSE Studiekeuze123 offers a means to provide general as well as more in-depth results. Our study is an attempt to show how policy makers in HEIs can make use of the NSE data to reinforce service quality, and promote student satisfaction and willingness to recommend.

For the OUNL in particular, the results offer a (service) quality-based approach to increase students’ satisfaction and propensity to recommend the OUNL. The most important service quality aspects and drivers of students’ satisfaction were provided by content and structure of the study, professors and lecturers, and academic guidance and counselling. Service quality of online education has become a major concern for most HEIs since they had to make a transition from on-campus to online education due to the Covid-19 pandemic. As online learning systems are expected to stay also after the Covid-19 pandemic is on its return, analyzing the service quality of the OUNL as a reputed online HEI can help traditional class-based HEIs getting their online learning systems on track. Generally, our results are congruent with those obtained in studies examining service quality in a face-to-face HE context. That is, corresponding service quality dimensions are relevant in both an online context and a face-to-face context. For example, in both surroundings competences, attitudes and didactic quality of academic staff are relevant academic service quality factors. Either face-to-face or virtual, teachers contribute significantly to their students’ learning processes (La Rotta et al., 2020). It is to be expected, however, that in an online environment academic staff needs to exploit other types of competences, attitudes and didactic approach compared to a face-to-face context. For example, in an offline context academics transfer (their) knowledge to students through often long lasting and busy class-based lectures, while in an online context academics primarily need to encourage and support students to gain knowledge themselves by studying, e.g. by offering students short but activating tasks that put them to work. The type of student-teacher interaction (teaching versus learning), therefore, is very different and requires other competences, attitudes and didactic quality from academics. The same holds for e.g. study facilities as a relevant service quality dimension in both online and offline environments. In an offline context physical resources (e.g. suitability of the classrooms, number of workplaces, and quality of study rooms) are fundamental in supporting the academic process. In an online context, however, these resources are expected to be less relevant or even not applicable since students are following an online program (off-campus) and are not likely to claim these services. On the other hand, however, a stable and easy to use electronic learning environment (ELO) is of core importance in an online environment (La Rotta et al., 2020; Uppal et al., 2018), while in a face-to-face context the interaction platform is more of a supportive nature. These potential differences in perceived service quality, however, need to be explored further and supported empirically. We will elaborate on this suggestion for further research in the next section.

### Limitations and directions for future research

Our study has some limitations providing avenues for more research in the future.

This study examined the service quality drivers for students’ satisfaction analyzing data from a single university in the Netherlands providing online education (the OUNL). Generalizations to a wider population, therefore, should be made with caution. Studiekeuze123, however, provides data for a lot of HEIs in the Netherlands and from 2010 up to now. This offers the opportunity for more representative follow-up studies examining the generalizability of the NSE measurement and the structural model in a wider HE context.

HE is a pure high-contact service requiring interpersonal contact over a long-term period to get an outcome (Sultan & Wong, 2012). In such circumstances, relationship quality is particularly suitable (Vieira et al., 2008). Therefore, when evaluating service quality in education, it is suggested to include (aspects of) relationship quality as a consequence of service quality. Though there is no consensus defining relationship quality, it can be defined as “the cognitive evaluation of business interactions by key individuals in the dyad, comparatively with potential alternative interactions” and includes several distinct but related components such as satisfaction, trust and commitment (Vieira et al., 2008). Although student satisfaction is found to be a primary antecedent to student loyalty, it is suggested that satisfaction alone is insufficient in generating loyalty (Bowden, 2011). Hennig-Thurau et al. (2001) and Sultan and Wong (2012, 2013), for example, found that perceived service quality positively affects trust in a HE context. Also, the relationship between service quality and commitment is suggested to be significantly positive (Hennig-Thurau et al., 2001). Both, trust and commitment are found to be contributing determinants, next to satisfaction, in generating loyalty (e.g., Garbarino & Johnson, 1999; Schijns et al., 2016), but have received limited attention in HE research (Bowden, 2011). Including additional components of relationship quality, such as trust and commitment, a more comprehensive model on service quality and its consequences can be developed and empirically tested in a HE context. A more comprehensive model can also be reached by including antecedents to service quality. Sultan and Wong (2012, 2013, 2014) suggest that (marketing) information and student’s past experiences with similar service encounters affect perceived service quality. As a result, a more comprehensive model contributes to a deeper understanding of how service quality, satisfaction and other relevant variables relate to each other and subsequently drive students’ loyalty.

The university included in this study is a public institution as most of the HEIs included in the NSE database. The NSE database, however, also includes a number of private HEIs. The results found in this study may differ for private HEIs. It may be worthwhile, therefore, to do a comparative study to explore potential differences in service quality and student satisfaction between HEIs in the public and private sector (Hasan et al., 2008).

Another type of comparative study can focus on potential differences in service quality perceptions between students following an online program and students following their program onsite. We concluded that corresponding service quality dimensions are relevant in both an online context and a face-to-face context. We suggest, therefore, that differences may not be found on the level of service quality dimensions, but on the level of items composing a service quality dimension. A group comparison (online versus face-to-face) complemented with an importance-performance analysis on item-level is expected to provide a better reflection of the characteristics of an online HEI and provide tools for HE that is developing distance learning.

The lack of background information limits gaining deeper insights. Including student’s background information in terms of e.g. gender, age etc., which is available to Studiekeuze123 but is not provided through the NSE database for privacy reasons, would help gaining deeper insights. Annamdevula and Bellamkonda (2016a: 457), for example, suggest that “age and gender play a major role in determining the different perceptions of students about the constructs investigated.” On the contrary, Bowden and Wood (2011) conclude that the role of gender doesn’t matter in the formation of student-university relationships.

The student-centered perspective on the quality of education was the central perspective of our study as in most studies on the quality of HEIs (Silva et al., 2017). As indicated in our introduction, however, quality of education is influenced by, and has an effect on, a number of stakeholders, such as: providers of resources (e.g. public and private funding bodies), users of outputs, i.e. graduates (e.g. employers), and employees of the HEI, e.g. academics and administrators. Studies from the point of view of stakeholders other than students, however, are scarce. Following for example Marinho and Poffo (2016), Smith et al. (2007) and Teeroovengadum et al. (2016) new studies could evaluate service quality in HEIs as perceived by e.g. academic staff, the management of the university, or the service department.

The NSE examines students’ service quality perceptions, students’ overall satisfaction and their willingness to recommend the HEI using one method, i.e. a questionnaire. The use of questionnaires in which respondents are asked to report their service quality perceptions, their overall satisfaction and their behavioural intentions is quite common in the social sciences and related fields since questionnaires have “particular advantages in terms of low expense, wide potential reach, and ease of administration”(Gorrell et al., 2011: 508). Common method bias is a type of bias often associated with questionnaire-based studies. Common method bias “refers to the situation where the method of data gathering itself introduces a bias, leading to spuriously elevated correlations between the concepts being measured” (Gorrell et al., 2011: 509). Thus, when using only one method measuring students’ service quality perceptions, their overall satisfaction and their willingness to recommend, common method bias is likely to be introduced. Future research in the field of service quality perceptions in HE should consider using different data gathering methods in an attempt to achieve a degree of data triangulation that further supports the validity of the NSE instrument.

To conclude, the NSE has become a standard in the Dutch HE context used by both HEIs and students (prospective as well as switching students) in their decision making. The scale has not been disseminated internationally, however, and adapting and applying the scale to other countries and/or cultures, therefore, is challenging. The current availability of the NSE questionnaire in German and English, besides Dutch, could facilitate international distribution of the questionnaire in that respect.

## Data Availability

Data are available on reasonable request.

## Appendix 1

**Table 6:** Formative constructs outer weights significance testing results

Formative constructs	Formative indicators	Outer weight	T statistics	P values	95% BCa confidence interval		Outer loading
Connection to professional field	…_01	0.293	5.873	0.000	0.195	0.390	0.903
	…_02	0.518	10.946	0.000	0.427	0.614	0.949
	…_03	0.279	5.793	0.000	0.184	0.372	0.874
General satisfaction	…_02	0.139	5.258	0.000	0.090	0.193	0.783
	…_03	0.040	1.678	0.093	−0.008	0.086	0.731
	…_04	0.102	4.378	0.000	0.057	0.151	0.717
	…_06	0.144	5.388	0.000	0.093	0.198	0.795
	…_07	0.115	4.750	0.000	0.068	0.162	0.756
	…_08	0.054	2.177	0.030	0.007	0.103	0.684
	…_09	0.151	6.413	0.000	0.105	0.197	0.776
	…_11	0.079	3.416	0.001	0.037	0.126	0.637
	…_12	0.162	6.182	0.000	0.114	0.215	0.795
	…_15	0.084	3.682	0.000	0.040	0.128	0.697
	…_16	0.090	4.224	0.000	0.049	0.134	0.687
	…_18	0.172	7.330	0.000	0.126	0.218	0.781
Acquired general skills	…_01	0.247	6.013	0.000	0.165	0.325	0.851
	…_03	0.339	7.519	0.000	0.254	0.430	0.891
	…_04	0.138	3.400	0.001	0.057	0.213	0.819
	…_05	0.169	5.117	0.000	0.106	0.234	0.741
	…_06	0.159	4.453	0.000	0.090	0.230	0.711
	…_07	0.164	3.878	0.000	0.080	0.246	0.830
Professors/Lecturers	…_01	0.243	6.540	0.000	0.166	0.312	0.870
	…_02	0.019	0.471	0.638	−0.063	0.101	0.857
	…_04	0.094	2.808	0.005	0.028	0.160	0.847
	…_05	0.252	6.334	0.000	0.176	0.329	0.913
	…_06	0.134	3.643	0.000	0.060	0.204	0.860
	…_07	0.209	5.665	0.000	0.138	0.281	0.900
	…_08	0.193	5.420	0.000	0.125	0.264	0.827
Information provided	…_02	0.281	5.856	0.000	0.188	0.377	0.831
	…_03	0.280	5.141	0.000	0.168	0.382	0.857
	…_04	0.269	5.493	0.000	0.169	0.363	0.846
	…_05	0.376	9.919	0.000	0.303	0.451	0.795
Content and structure of study	…_01	0.177	6.850	0.000	0.127	0.227	0.836
	…_02	0.132	4.809	0.000	0.078	0.186	0.823
	…_03	0.031	1.485	0.138	−0.011	0.072	0.658
	…_04	0.210	6.905	0.000	0.151	0.270	0.842
	…_05	0.099	3.991	0.000	0.051	0.150	0.762
	…_06	0.083	3.124	0.002	0.031	0.134	0.782
	…_07	0.212	8.123	0.000	0.162	0.265	0.797
	…_09	0.181	6.897	0.000	0.130	0.233	0.816
	…_10	0.034	1.196	0.232	−0.022	0.088	0.723
	…_11	0.089	3.277	0.001	0.034	0.141	0.720
Quality care	…_01	0.331	8.068	0.000	0.249	0.412	0.878
	…_02	0.084	1.696	0.090	−0.011	0.183	0.856
	…_03	0.263	4.468	0.000	0.148	0.380	0.915
	…_04	0.432	9.756	0.000	0.341	0.517	0.919
Academic guidance counselling	…_04	0.261	5.323	0.000	0.163	0.358	0.916
	…_05	0.460	9.045	0.000	0.357	0.555	0.952
	…_06	0.357	9.079	0.000	0.279	0.435	0.904
Study facilities	…_01	0.276	5.770	0.000	0.181	0.369	0.809
	…_06	0.236	4.264	0.000	0.126	0.343	0.871
	…_07	0.376	7.169	0.000	0.272	0.475	0.891
	…_08	0.278	5.206	0.000	0.174	0.384	0.851
Study load	…_01	0.191	3.319	0.001	0.080	0.302	0.850
	…_02	0.232	3.764	0.000	0.108	0.354	0.861
	…_04	0.255	4.738	0.000	0.150	0.360	0.830
	…_05	0.393	8.656	0.000	0.302	0.481	0.850
	…_06	0.117	2.098	0.036	0.005	0.222	0.790
Testing and assessment	…_01	0.280	6.981	0.000	0.200	0.358	0.849
	…_02	0.275	5.240	0.000	0.174	0.376	0.920
	…_04	0.206	3.767	0.000	0.095	0.314	0.918
	…_05	0.351	8.198	0.000	0.266	0.434	0.906
	…_06	0.011	0.349	0.727	−0.049	0.071	0.174
Challenging character of study	…_01	0.480	15.194	0.000	0.416	0.540	0.911
	…_02	0.208	6.041	0.000	0.140	0.274	0.821
	…_03	0.248	6.598	0.000	0.174	0.321	0.880
	…_04	0.234	8.951	0.000	0.184	0.287	0.742
Acquired scientific skills	…_01	0.409	10.164	0.000	0.326	0.484	0.891
	…_02	0.219	4.287	0.000	0.121	0.320	0.888
	…_04	0.129	2.351	0.019	0.026	0.242	0.843
	…_05	0.127	2.460	0.014	0.023	0.228	0.843
	…_07	0.257	4.737	0.000	0.152	0.361	0.876

## Appendix 2

**Table 7:** Three critical service quality aspects covered by performance-based service quality models, developed specifically for application in HE context

	PHEd-scale	HEdPERF-scale	HESQUAL-scale	NSE-scale resp. this study
	8 dimensions: 1. Dependability; 2. Effectiveness; 3. Capability; 4. Efficiency; 5. Competencies; 6. Assurance; 7. Unusual situation management; 8. Semester-syllabus.	6 dimensions: 1. Non-academic aspects; 2. Academic aspects; 3. Reputation; 4. Access; 5. Program issues; 6. Understanding.	5 dimensions: 1. Administrative quality; 2. Physical environment quality; 3. Core educational quality; 4. Support facilities quality; 5. Transformative quality.	19 dimensions resp. 12 dimensions (see Table 1)
	Includes 26 service quality items.	Includes 41 service quality items.	Includes 48 service quality items.	Includes 89 resp. 60 service quality items.
Academic service quality	- Teaching quality: competent lecturers and professors; - Availability of lecturers; - Course development: program and content; - Teacher-student relationships.	- Knowledgeable in course content; - Caring and courteous staff; - Responding staff; - Feedback on progress.	- Attitudes and behaviors of academics; - Curriculum; - Pedagogy; - Competence of academics; - Transformative quality (e.g., developing general, scientific and professional skills).	- The content, structure, and cohesion of the study program; - Acquirement of general skills; - Acquirement of scientific skills; - Didactic quality of Professors and Lecturers.
Administrative service quality	- Skills and abilities of administrative and support staff in providing services; - Effective responses to students’ requests; - Efficient and effective admission procedures; - Their relationships with students; - Providing adequate information; - Process of administering and delivering education.	- Duties carried out by non-academic staff; - Caring and individualized attention; - Efficient/prompt dealing with complaints; - Staff knowledgeable of systems/procedures.	- Attitudes and behaviors of administrative staff members; - Administrative processes (efficient, effective, structured, transparent).	- Information provided by and responsiveness of the institution, e.g. about study progress; - Timely publication of programme schedules and schedule changes.
Facilities service quality	- Library facilities; - Entertainment facilities; - Career counselling; - Transport facilities; - Dining facilities; - Access to e.g., computers, workshops, seminars, and conferences.	- Counselling services; - Health services; - Hostel facilities and equipment; - Academic facilities; - Recreational facilities; - Internal quality programmes;	- Support infrastructure (e.g., library infrastructure); - Learning setting (e.g., teaching tools and equipments); - General infrastructure (e.g., appearance of buildings and grounds) - Support facilities (e.g., IT, photocopy and printing facilities).	- Study supervision (Academic guidance/counselling); -Study facilities, e.g. digital learning environment/system; access to digital library; - IT facilities; -Suitability of classrooms/meeting rooms.
Literature source:	E.g., Sultan and Wong (2010b, 2012, 2013, 2014)	E.g., Abdullah (2006)	E.g., Teeroovengadum et al. (2016, 2019)	E.g., Brenders (2013); Studiekeuze123 (2019)
